# β2-Adrenergic receptor agonists ameliorate the adverse effect of long-term pyridostigmine on neuromuscular junction structure

**DOI:** 10.1093/brain/awz322

**Published:** 2019-10-21

**Authors:** An E Vanhaesebrouck, Richard Webster, Susan Maxwell, Pedro M Rodriguez Cruz, Judith Cossins, James Wickens, Wei-wei Liu, Hakan Cetin, Jonathan Cheung, Hayley Ramjattan, Jacqueline Palace, David Beeson

**Affiliations:** 1 Neurosciences Group, Weatherall Institute of Molecular Medicine, John Radcliffe Hospital, University of Oxford, Oxford, OX3 9DS, UK; 2 Department of Clinical Neurology, John Radcliffe Hospital, University of Oxford, Oxford, OX3 9DU, UK; 3 Chemistry Research Laboratory, Department of Chemistry, University of Oxford, Oxford, OX1 3TA, UK; 4 Paediatric Neurology, John Radcliffe Hospital, University of Oxford, Oxford, OX3 9DU, UK

**Keywords:** myasthenia, congenital myasthenic syndromes, AChR deficiency, β2-adrenergic, acetylcholinesterase inhibitor

## Abstract

Acetylcholine receptor deficiency is the most common form of the congenital myasthenic syndromes, a heterogeneous collection of genetic disorders of neuromuscular transmission characterized by fatiguable muscle weakness. Most patients with acetylcholine receptor deficiency respond well to acetylcholinesterase inhibitors; however, in some cases the efficacy of acetylcholinesterase inhibitors diminishes over time. Patients with acetylcholine receptor deficiency can also benefit from the addition of a β2-adrenergic receptor agonist to their medication. The working mechanism of β2-adrenergic agonists in myasthenic patients is not fully understood. Here, we report the long-term follow-up for the addition of β2-adrenergic agonists for a cohort of patients with acetylcholine receptor deficiency on anticholinesterase medication that demonstrates a sustained quantitative improvement. Coincidently we used a disease model to mirror the treatment of acetylcholine receptor deficiency, and demonstrate improved muscle fatigue, improved neuromuscular transmission and improved synaptic structure resulting from the addition of the β2-adrenergic agonist salbutamol to the anticholinesterase medication pyridostigmine. Following an initial improvement in muscle fatiguability, a gradual decline in the effect of pyridostigmine was observed in mice treated with pyridostigmine alone (*P <* 0.001). Combination therapy with pyridostigmine and salbutamol counteracted this decline (*P <* 0.001). Studies of compound muscle action potential decrement at high nerve stimulation frequencies (*P <* 0.05) and miniature end-plate potential amplitude analysis (*P <* 0.01) showed an improvement in mice following combination therapy, compared to pyridostigmine monotherapy. Pyridostigmine alone reduced postsynaptic areas (*P <* 0.001) and postsynaptic folding (*P <* 0.01). Combination therapy increased postsynaptic area (*P <* 0.001) and promoted the formation of postsynaptic junctional folds (*P <* 0.001), in particular in fast-twitch muscles. In conclusion, we demonstrate for the first time how the improvement seen in patients from adding salbutamol to their medication can be explained in an experimental model of acetylcholine receptor deficiency, the most common form of congenital myasthenic syndrome. Salbutamol enhances neuromuscular junction synaptic structure by counteracting the detrimental effects of long-term acetylcholinesterase inhibitors on the postsynaptic neuromuscular junction. The results have implications for both autoimmune and genetic myasthenias where anticholinesterase medication is a standard treatment.

## Teaser

The efficacy of anticholinesterase medication may diminish over time. Vanhaesebrouck *et al.* use an animal model of acetylcholine receptor deficiency syndrome to show that beta-2 adrenergic receptor agonists ameliorate the damage to neuromuscular junction structure caused by prolonged anticholinesterase medication, and thereby counteract the decline in efficacy.

## Introduction

Acetylcholinesterase inhibitors are commonly used as symptomatic treatment in many forms of myasthenia (Engel, 2007; Mehndiratta *et al.*, 2014). However, several studies on rodent muscle in the 1970s suggested that long-term anticholinesterase therapy might have an adverse effect on motor end-plate postsynaptic structures and thus on neuromuscular transmission (Engel *et al.*, 1973; Gillies and Allen, 1977). The congenital myasthenic syndromes (CMS) are a heterogeneous group of hereditary disorders of neuromuscular transmission characterized by fatiguable muscle weakness (reviewed in Beeson, 2016; Engel, 2018). In the UK, acetylcholine receptor (AChR) deficiency due to mutations in the AChR ε-subunit gene (*CHRNE*) is the most common form of congenital myasthenic syndrome (Parr *et al.*, 2014). As its name implies, in this syndrome there is a marked reduction in the number of AChRs on the postsynaptic membrane, although clinical severity is variable. In general there is a good response to anticholinesterase medication, but in a number of cases we have observed that the efficacy of acetylcholinesterase inhibitors clearly diminishes over time (which may also be seen in myasthenia gravis; Drachman, 2012). In some cases, despite optimized therapy with pyridostigmine and 3,4-diaminopyridine (3,4-DAP), patients may remain severely disabled (Rodriguez Cruz *et al.*, 2015).

β2-Adrenergic (receptor) agonists, such as salbutamol and ephedrine, have been found to have a marked therapeutic benefit for patients who harbour mutations in the agrin-induced LRP4-MuSK-DOK7 AChR clustering pathway (Schara *et al.*, 2009; Ben Ammar *et al.*, 2010; Lashley *et al.*, 2010; Liewluck *et al.*, 2011), which is crucial for the formation and maintenance of the mature neuromuscular junction structure (Burden *et al.*, 2018), suggesting that the β2-adrenergic agonists provide a compensatory mechanism to partially mitigate disruption of this pathway. It is proposed that maturation and maintenance of the neuromuscular synapse is regulated through signalling from MuSK that counteracts a destabilizing effect of neurotransmission itself on synaptic structure (Lin *et al.*, 2005; Kummer *et al.*, 2006). Acetylcholinesterase inhibitors, which increase the number of AChRs activated by each quanta (containing acetylcholine) released by a nerve impulse, prolong neurotransmission, and thus long-term they may act to destabilize the fine structure of the synapse (Lin *et al.*, 2005; Kummer *et al.*, 2006). Therefore, it is proposed that β2-adrenergic agonists would be beneficial for AChR deficiency cases on long-term treatment with pyridostigmine (Beeson, 2016), with both a case report (Sadeh *et al.*, 2011) and a prospective quantitative study (Rodriguez Cruz *et al.*, 2015) providing support.

Here, we report the longer term quantitative outcome of adding a β2-adrenergic agonist to treat AChR deficiency patients on anticholinesterase inhibitors. The role of the β2-adrenergic receptor for anabolic action on skeletal muscle and for a shift from slow to fast muscle fibre type profile is well known (Lynch and Ryall, 2008), but its function at the neuromuscular junction is poorly understood. To investigate what occurs *in vivo* at the neuromuscular junction, we used a mouse model of AChR deficiency syndrome that accurately reflects the human condition (Cossins *et al.*, 2004). Our study mimics the treatment of AChR deficiency syndrome patients (Rodriguez Cruz *et al.*, 2015), and demonstrates the effects of pyridostigmine and salbutamol on neuromuscular structure and function.

## Materials and methods

### Patient cohort and clinical assessment

Patients were followed up at the UK National Diagnostic and Advisory Congenital Myasthenia Service in Oxford. Clinical details and objective strength measurements were recorded routinely at each visit. All patients harboured recessive ‘loss of expression’ mutations in *CHRNE*. Strength measurements included the quantitative myasthenia gravis (QMG) severity score (Jaretzki *et al.*, 2000) before and after the initiation of treatment with β2-adrenergic agonists. Components for ptosis, double vision, facial weakness, swallowing, and speech disturbances were based on clinical interpretation. Components for arm raise time, leg raise time, grip strength, head lift time, and forced vital capacity were based on objective, continuous values assigned to a range (e.g. for arm raise time: 0 ≥ 240 s, 1 = 91–240 s, 2 = 11–90 s, and 3 ≤ 10 s). The QMG severity score is composed of 13 components with each scored from 0 (normal) to 3 (severe weakness). β2-Adrenergic agonist therapy was only begun once therapy with acetylcholinesterase inhibitor and/or 3,4-diaminopyridine was optimized. Dosage of salbutamol or ephedrine was dependent on body weight and tolerability. Written informed consent for analysis and publication of genetic and clinical data were obtained for all patients (Oxfordshire Research Ethics Committees B, 04.OXB.017 and C, 09/H0606/740).

### Model mouse breeding, genotyping and drug treatment

A transgenic mouse model of AChR deficiency was used, as generated by Cossins *et al.* (2004). Model mice (hγ^+^mε^−/−^) constitutively express low levels of the human AChR γ-subunit along the length of muscle fibres under a skeletal muscle α-actin promotor in an AChR ε-subunit ‘knock-out’ background (*Chrne*^tm1Jrs^) (Missias *et al.*, 1997). Their genetic background is C57BL/6. Mice breeding and genotyping was performed as described previously (Cossins *et al.*, 2004). Salbutamol sulphate (Sigma-Aldrich) and pyridostigmine (Sigma-Aldrich) for treatment of mice were dissolved in drinking water. Serum levels of salbutamol were analysed by liquid chromatography-electrospray-tandem mass spectrometry.

### Muscle fatiguability measured by the inverted screen test

The inverted screen or hanging test, as first described by Kondziella, is commonly used to reliably test muscle fatigue in myasthenic mice (Kondziella, 1964; Kusner *et al.*, 2015). Mice were placed in the centre of a 50 cm^2^ wire mesh screen. The screen was rotated to the inverted position. The time that mice held on the screen was recorded. The cumulative time was recorded for three consecutive attempts without resting in between. To mirror the severity of disease in our patient cohort, model mice with a cumulative hang time exceeding 4 min during one of the first three inverted screen tests were excluded from the study.

### Recording of compound muscle action potential decrement following repetitive nerve stimulation

Repetitive nerve stimulation was performed, as described previously (Webster *et al.*, 2013). Anaesthesia was induced using intraperitoneal fentanyl 0.1 mg/kg and fluanisone 3 mg/kg (Hypnorm®, Vetpharma Ltd.) and maintained using inhaled isoflurane (1.25–1.75%)/O_2_. Mouse rectal temperature was maintained between 37°C and 38°C. The sciatic nerve was stimulated at the level of the hip. Compound muscle action potentials (CMAPs) were recorded from gastrocnemius muscles (Dual bio amp/stimulator and Powerlab 4/25, AD Instruments). A train of minimum 10 stimuli was applied. The amplitude of the CMAP decayed maximally by the 10th response in model mice, and therefore decrement was assessed by comparison of first and 10th stimulus in a train (Klooster *et al.*, 2012; Sanders *et al.*, 2014) (pClamp 9, Molecular Devices). Decrement was analysed at varying stimulation frequencies, ranging from 1 to 100 Hz (Wu *et al.*, 2016). Results of three test series were averaged.

### End-plate potential recordings in phrenic nerve-diaphragm preparations

Following euthanasia, phrenic nerve/hemi-diaphragm preparations were dissected and bathed in Krebs solution, containing 2.5 mM CaCl_2_ and bubbled with 95% O_2_/5% CO_2_ (Cossins *et al.*, 2004). μ-Conotoxin GIIIB (2.5 μM, Peptide Institute Inc.) was added to the bath for 30 min to block muscle contractions. The excess unbound toxin was washed out before recordings began. Recordings were made at 22–23°C.

The phrenic nerve was pulled into a suction electrode, which was coupled to a pulse generator, with an associated stimulus isolation unit (GRASS instruments S48 square pulse stimulator). Recording electrodes were connected to an Axoclamp 900A amplifier (Molecular Devices). Data signals passed through a Humbug 50 Hz noise eliminator (Quest Scientific via Digitimer). Signals were continuously digitized at 10 kHz sampling rate and filtered at 2 kHz, using Axon Digidata 1322A interface, controlled by pClamp 10 software (Molecular Devices). Depolarizations at the end-plate were recorded intracellularly using a single borosilicate glass micropipette electrode. Electrodes were pulled by a programmable P-97 microelectrode puller (Sutter Instruments) and filled with 3 M KCl (10–30 MΩ). The recording electrode was positioned above end-plate regions, as visualized by a stereomicroscope (Olympus BX51WI) under micromanipulator control (Scientifica).

Impalement adjacent to an end-plate was indicated by fast rise time of miniature end-plate potentials (mEPPs), defined as <3 ms in model mice. To evoke an EPP, the phrenic nerve was stimulated via two silver-wire electrodes. Pulses of supramaximal intensity were used (typically 3–6 V). If the membrane potential depolarized beyond −55 mV, the recording was abandoned for that end-plate. Recordings or part of recordings with an unstable or drifting membrane potential were excluded from analysis. Ten to 30 end-plates per animal were sampled.

Each mEPP and EPP was detected via template or threshold searching in Clampfit 10 software. Analysis was performed blinded to treatment group. All mEPP and EPP amplitude measurements were adjusted for deviation of a resting membrane potential of −80 mV (Katz and Thesleff, 1957). Mean mEPP amplitude was derived from averaging 20–40 mEPPs per end-plate. Mean EPP amplitude per end-plate was derived from averaging a train of 20 EPPs evoked at 1 Hz.

Mean quantal content (*m*) was calculated per end-plate, by dividing the mean amplitude of EPP (stimulated at 1 Hz) by the mean amplitude of mEPP, using the formula:

(1) $$m=\text{mean}\left({\text{AMP}}_{\text{EPP}(\text{corrected})}\right)/\text{mean}\left({\text{AMP}}_{\text{mEPP}}\right)$$

where *AMP_EPP_*_(__*corrected*__)_ is the corrected amplitude of the EPP and *AMP_mEPP_* is the amplitude of the mEPP. In the above calculation, EPPs (evoked at 1 Hz) were corrected for non-linear summation, using the formula:

(2) AMPEPP(corrected)=AMPEPP(measured)(1−0.8AMPEPP(measured))E

In the above formula, *E* is the driving force and was assumed to be 80 mV, and 0.8 was arbitrarily used as the correction factor for mouse end-plates (Linder and Quastel, 1978; McLachlan and Martin, 1981). For experiments of EPP rundown, trains of EPPs were recorded at a range of increasing nerve stimulation frequencies (0.5 to 100 Hz).

### Morphological analysis of neuromuscular junctions

Extensor digitorum longus, soleus, and diaphragm muscles were dissected in Krebs buffer, bubbled with 95% O_2_/5% CO_2_. Muscles were pinned out on blocks of Sylgard™. Immediately following dissection (to maximally preserve neuromuscular junction morphology), tissues were fixed for 30 min in phosphate-buffered saline (PBS) containing 1% formaldehyde. Muscle tissue from wild-type mice was included as a positive staining control.

Then, muscles were incubated overnight at 4°C with α-bungarotoxin Alexa Fluor® 594 conjugate (ThermoFisher Scientific, B13423, 1:150) and fasciculin-2 Alexa-Fluor® 488 conjugate (Invitrogen, custom-made, 1:500) diluted in PBS (Krejci *et al.*, 2006). Tissues were washed and teased out in single fibres for extensor digitorum longus and soleus muscles, and whole-mounted for diaphragm muscles.

Fluorescence was observed using an Olympus IX71 wide-field fluorescence microscope. Identical settings were used for image capture, using Simple PCI software (Digital Pixel). A minimum of 20 pictures from randomly selected areas was taken using a 40× objective. Images were later quantitatively analysed in a blinded fashion. Areas, intensities and fragmentation of neuromuscular junction morphology were measured using a custom macro made with Fiji software (Schindelin *et al.*, 2012). AChR staining was faint in model mice. In particularly for soleus and diaphragm muscles, synaptic AChR staining was sometimes difficult to distinguish from extrasynaptic AChR staining, and occasionally only a rim or no clear AChR synaptic area (assigned value = 0) could be measured.

### Muscle fibre size and fibre type identification

Extensor digitorum longus and soleus muscles were frozen in isopentane cooled in liquid nitrogen. Transverse sections were taken of the middle part of the muscle using a Leica CM1900 cryostat at a thickness of 10 μm. Sections were mounted on slides and stored at –20°C until further processing. Primary antibodies used for fibre typing were: mouse anti-myosin slow (type I) and fast (type IIa and IIb) heavy chain monoclonal antibodies (NovoCastra, NCL-MHCs and NCL-MHCf, 1:40). In addition, rabbit anti-laminin antibody (Sigma-Aldrich, L9393, 1:200) was used to delineate the periphery of each muscle fibre. Secondary antibodies coupled to fluorochrome Alexa Fluor® 488 and 594 were used to visualize primary antibody staining. Slides were labelled with a code, and images were taken blinded to treatment group. Images were captured at ×10 magnification with a wide-field fluorescence microscope (Olympus IX71) and Simple PCI software (Digital Pixel). Slides were analysed in a blinded manner. A Fiji macro was made for semi-automated analysis of fibre size and fibre type proportions. An average of 250 muscle fibres per muscle per animal were examined.

### Electron microscopy of end-plate regions

Extensor digitorum longus tissue was dissected following euthanasia, pinned at resting length and immersed in fixative within 30 min. Tissue was fixed in 2.5% glutaraldehyde/ 2% PFA in 0.1 M sodium cacodylate buffer (pH 7.2) for 1 h, then stored at 4°C until further processing.

Sections of ~100 μm were cut in the longitudinal plane, using a vibratome (Leica VT1200 S Vibratome). Samples were post-fixed in osmium tetroxide in 0.1 M cacodylate buffer, then washed and incubated in aqueous uranyl acetate (Shanks *et al.*, 2017). Samples were dehydrated in a graded ethanol series and infiltrated with epoxy resin, using a Leica EM AMW automatic microwave tissue processor (Leicam Microsystems Inc.). Individual tissue pieces were transferred to BEEM® capsules filled with fresh resin, then polymerized.

Blocks were sectioned using a Leica UC7 ultramicrotome. First, semi-thin sections (500 nm) were taken using a glass knife and regions with nerve terminals on toluidine staining were selected for ultra-thin sectioning (90 nm). Ultra-thin sections were taken with a diamond knife (Diatome), placed on 200-mesh copper grids and post-stained with lead citrate. Images of sections of blocks were taken, in a random fashion, using a FEI Tecnai 12 transmission electron microscope. A minimum of 30 end-plate regions per group were captured and analysed. Images were taken by a second investigator (S.M.) who was blinded to the treatment group. A code (with no information regarding treatment group) was assigned to each image by the second investigator. Images were later analysed by the primary investigator (A.V.), blinded to treatment group. After complete analysis, codes were unblinded. An end-plate region was defined as a nerve terminal and its associated postsynaptic region. All morphometric parameters of end-plate regions were quantitatively analysed according to Engel and Santa (1971), i.e. presynaptic length, postsynaptic length (including length of folds) and folding index (postsynaptic length divided by presynaptic length). For this purpose, we used Fiji to first set the scale of each image. Then, we outlined the pre- and postsynaptic membrane of each end-plate with Fiji ‘free hand line’ tool, with length of the drawn line automatically measured by Fiji software.

### Statistical analysis

Graphic design and statistical analysis was performed with GraphPad Prism version 7 (GraphPad Software) and SPSS version 25.0 (IBM SPSS). Because of the longitudinal, hierarchical and multivariate nature of the data, a linear mixed model was used to compare treatment groups. Groups were compared using the restricted maximum likelihood method. Model reductions were based on likelihood ratio tests of maximum likelihood estimates. In brief, treatment group, time, stimulation frequency, stimulus number, muscle, batch and/or their interaction were entered as fixed effects in the model, as appropriate. Random effects included the intercept for each subject, except for ultrastructural analysis. Data were checked for normality and transformed if necessary to meet linear mixed model assumptions. The main comparison was between combination therapy (pyridostigmine plus salbutamol) and pyridostigmine monotherapy, as based on our *a priori* hypothesis. Additional ‘informative’ comparisons were made between untreated and monotherapy groups (either pyridostigmine or salbutamol). *P*-values (two-tailed) were obtained from the model, using the Tukey method. Levels of statistical significance were set at threshold *P* < 0.05.

Data in graphs, tables and text are presented as unadjusted means and 95% confidence intervals (95% CI), i.e. obtained from descriptive statistics, unless otherwise stated. Statistical significance presented in graphs, tables and text represents *P-*values obtained following fitting the data in the mixed model, as described above.

### Data availability

The data that support the findings of this study are available from the corresponding author, upon reasonable request. 

## Results

### Long-term follow-up of the addition of salbutamol or ephedrine to AChR deficiency patients on pyridostigmine

**Figure 1:**
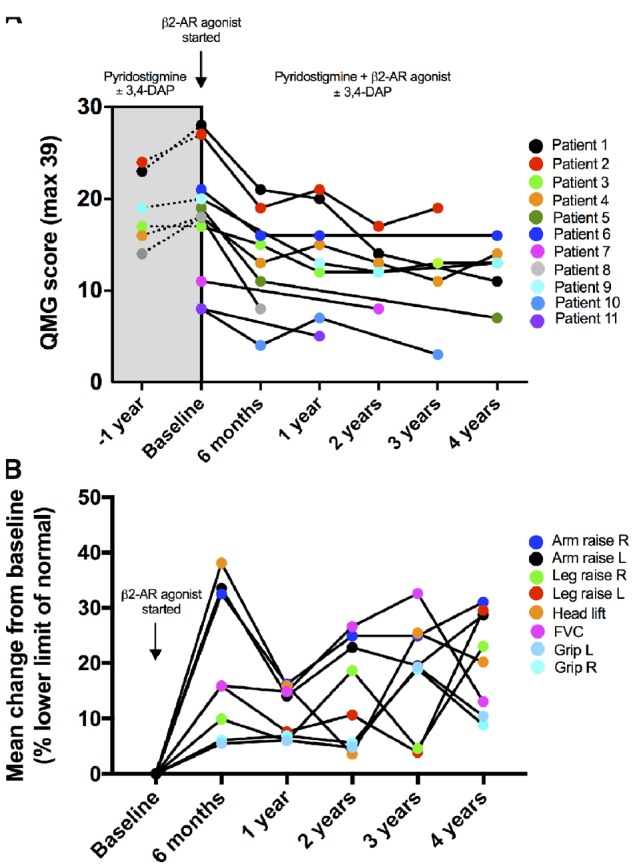
**Quantitative assessment of the response of AChR deficiency patients to the addition of salbutamol or ephedrine to their medication.** (**A**) Change in individual QMG severity scores (max = 39) over a 4-year follow-up period. In patients with AChR deficiency, already on optimized therapy with pyridostigmine ± 3,4-diaminopyridine (3,4-DAP), β2-adrenergic agonists (β2-AR, typically salbutamol) were introduced, often following clinical deterioration. (**B**) Change in percentage of the lower limit of normal (minimum required to achieve 0 in the QMG scale) for the individual quantitative components that contribute to the QMG severity score. FVC = forced vital capacity.

Previously we reported the response of a cohort of six patients with severe AChR deficiency due to mutations in *CHRNE* on optimum medication with pyridostigmine and 3,4-diaminopyridine, who were additionally given salbutamol or ephedrine medication for 6 months (Rodriguez Cruz *et al.*, 2015). Here we report the longer term follow-up (for up to 4 years) of these cases and a further five additional cases, making a case series of 11 patients for whom we were able to assess strength measurements including the QMG severity score (Jaretzki *et al.*, 2000) before and after the initiation of treatment with β2-adrenergic agonists (Fig. 1). All cases were on long-term anticholinesterase medication. Details on *CHRNE* mutations and medication dosage are given in Supplementary Table 1. The QMG severity score is not suitable for assessing disease severity in children and thus our cohort is restricted to adults. In some cases a QMG severity score was available a year prior to starting the β2-adrenergic agonist therapy. Mean QMG severity scores (a higher score indicates greater disability) improved from baseline to 6 months of pyridostigmine plus β2-adrenergic agonist combination therapy, i.e. from 17.73 (95% CI 13.25–22.2) to 13.38 (95% CI 8.668–18.08) (*P <* 0.001) (Fig. 1A). At 4 years follow-up on treatment, the mean QMG severity score was 12.33 (95% CI 9.10–15.56). Comparison of QMG severity scores at 6 months and 4 years showed sustained clinical benefit over time with a further decrease (albeit not statistically significant) in the QMG severity score. The mean change from baseline as a percentage of the lower limit of normal for the individual components of the QMG severity score that have a quantitative basis are shown in Fig. 1B. This analysis of the QMG components again emphasizes the sustained benefit of combination therapy. Thus, the benefit of adding salbutamol into the treatment regimen for this cohort of patients is maintained over a prolonged period.

### Protocol design for the study of the response to treatment in a mouse model for AChR deficiency

We performed a study of treatments on a mouse model of AChR deficiency that was aimed to replicate the experience of our patient cohort. Our mouse model (Cossins *et al.*, 2004) was designed to mirror the human condition, with the cDNA encoding the human AChR γ-subunit constitutively expressed at low levels along the length of the muscle fibres and incorporated into end-plate AChR at markedly reduced levels. The treatment protocol used model mice from 12 weeks of age, as expression of the mouse AChR γ-subunit is turned off by postnatal Day 21, and by 12 weeks levels of endogenous mouse AChR γ-subunit are undetectable. As the human γ-subunit AChR transgene was inserted randomly into the X-chromosome of model mice, only male model mice were included in the trial (Carrel and Willard, 2005). Ten to 13 mice per treatment group were included. There were four treatment groups: (i) the first group remained untreated for the whole duration of the trial; (ii) the second group received pyridostigmine from Weeks 2 to 12; (iii) the third group received no treatment between Weeks 2 and 8, then salbutamol was added from Weeks 8 to 12; and (iv) the fourth group received pyridostigmine from Weeks 2 to 12, to which salbutamol was added from Weeks 8 to 12. The term ‘combination therapy’ was used to refer to treatment with pyridostigmine and salbutamol in combination. In the first week, all groups were untreated. Model mice were allocated to one of the four treatment groups according to a computer-generated randomization list. Body weight and muscle fatiguability (by the inverted screen test) were tested twice weekly. Decrement of CMAPs was assessed prior to each treatment change and at the end of the trial. At the end of the trial (Week 12) mice were sacrificed, and neuromuscular transmission and structure of neuromuscular junctions, as well as muscle fibre size and type were assessed.

Pilot experiments were performed to determine the doses that should be used in the treatment study. Based on a first pilot study, salbutamol at a dose of 45 mg/kg/day (given via drinking water) was chosen to ensure serum salbutamol concentrations fell within the expected therapeutic window of 3–20 ng/ml extrapolated from human studies (Chik *et al.*, 2009). A similar pilot study showed pyridostigmine at a dose of 14 mg/kg/day was the most effective dose at increasing hang time in the inverted screen test. Serum salbutamol concentrations were confirmed by mass spectrometry at the end of the actual treatment protocol following 4 weeks of salbutamol treatment. Serum was collected from male littermates, housed in the same cage as model animals. Median serum level of salbutamol was 10.9 ng/ml (range 4.2–143.4 ng/ml, *n* = 7).

### Salbutamol added to pyridostigmine reduces fatiguable muscle weakness

**Figure 2:**
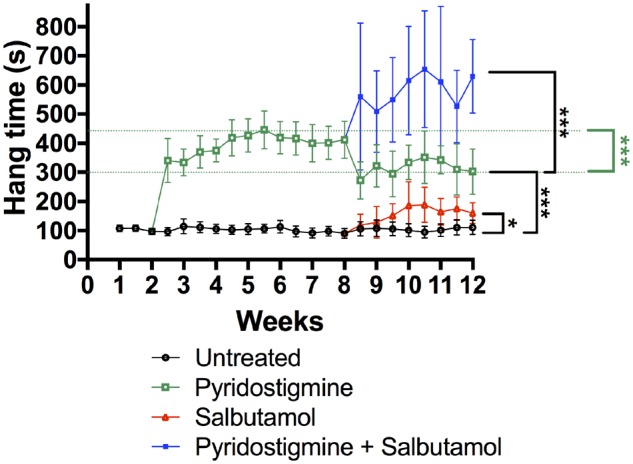
**Improvement of muscle fatiguability for the AChR deficiency disease model during treatment protocol, as assessed by the inverted screen test.** Fatiguable weakness was assessed twice weekly during the treatment trial of mice in four treatment groups: untreated (black), pyridostigmine alone (green), salbutamol alone (red), and combination therapy of pyridostigmine plus salbutamol (blue). Datapoints and error bars represent group means and 95% CI. The black significance bars compare overall group results between the period of 8.5 to 12 weeks. The green significance bar compares maximum value with value at last time point (under pyridostigmine treatment). *n =* 10–13 mice per group; *P < 0.05, ***P < 0.001 obtained from linear mixed model.

Model mice gained 5% of body weight during the trial (Supplementary Fig. 1). A temporary decrease in body weight was observed following procedures under general anaesthesia. Body weight was unaffected by drug treatment. The results of the measurement of muscle fatiguability, as determined by the inverted screen test, during the duration of the treatment trial are shown in Fig. 2. As expected, model mice hang for a shorter time on the inverted screen (mean <2 min, see Fig. 2) than wild-type mice (median 19.1 min, range 16.1–43.1, *n =* 3). Untreated model mice did not worsen in strength over the course of the trial. Model mice treated with pyridostigmine alone showed a marked improvement on the inverted screen test, compared to untreated mice (*P <* 0.001). However, over time this improvement gradually became less robust in mice treated with pyridostigmine alone, with a significant reduction in hang time from peak value (5.5 weeks) to value at last time point (12 weeks) (*P <* 0.001). Treatment with salbutamol alone also resulted in a modest improvement of performance in model mice, compared to untreated mice (*P* = 0.025). Mice treated with combination therapy (pyridostigmine plus salbutamol) performed significantly better than mice treated with pyridostigmine alone (*P <* 0.001). This improvement began to be apparent shortly after starting salbutamol addition. There was no decrease in performance during the course of combined treatment (in contrast to animals treated with pyridostigmine alone), with a continuing improvement over time noted.

### Salbutamol added to pyridostigmine reduces decrement at high nerve stimulation frequencies

**Figure 3:**
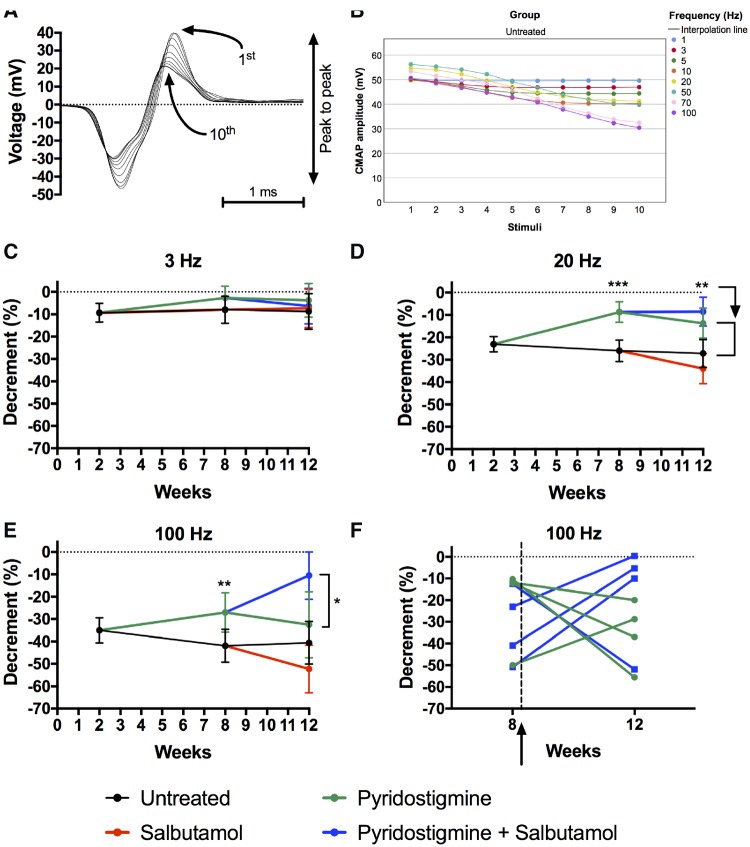
**Decrement in model mice during the treatment protocol.** (**A**) Example trace of train of superimposed CMAPs in gastrocnemius muscle following repetitive stimulation of sciatic nerve. (**B**) The amplitude of the CMAP decreases with higher stimulation frequencies in model mice (1–100 Hz) and the amplitude of the CMAP decays maximally by the 10th response. Datapoints represent means of (untreated) model mice, with confidence interval omitted for clarity. (**C**–**E**) Repeated assessment of CMAP decrement (comparing peak-to-peak amplitudes between the 10th and the first response) during treatment trial at 3 (**C**), 20 (**D**) and 100 Hz (**E**). Datapoints and error bars represent adjusted group means (i.e. controlled for imbalance in baseline values at Week 2) and 95% CI (*n =* 10–13 mice per group for 3 and 20 Hz, *n =* 4–5 mice per group for 100 Hz; *P < 0.05, **P < 0.01, ***P < 0.001 obtained from linear mixed model). (**F**) Line graph, showing changes of decrement (at 100 Hz, comparing 10th with first response) over time for individual animals either treated with pyridostigmine or treated with combination therapy. The arrow indicates the time that salbutamol was added to pyridostigmine. Significance between combination therapy and pyridostigmine monotherapy was absent when analysing decrement at earlier stimuli, i.e. when comparing the fourth with the first response.

We examined whether differences in muscle fatiguability coincided with differences in neurotransmission function by examining decrement in amplitudes of CMAPs (Fig. 3A). The amplitude of the CMAP decayed maximally by the 10th response in model mice (Fig. 3B). Increasing nerve stimulation frequencies were used to maximize decrement (Fig. 3B). Mean decrement in model mice was less than 10% at low nerve stimulation frequencies (3 Hz), but approached 25% at moderate nerve stimulation frequencies (20 Hz) and 40% at high nerve stimulation frequencies (100 Hz). Mean decrement in wild-type mice was less than 3.6% irrespective of frequency. Figure 3C–E shows decrement results of model mice during the treatment trial at low (3 Hz), moderate (20 Hz) and high stimulation frequencies (100 Hz). Pyridostigmine treatment of the model mice significantly reduced decrement at all stimulation frequencies above 5 Hz mid-trial (*P <* 0.05). No effect of pyridostigmine was detected at stimulation frequencies less than 5 Hz, presumably due to mild decrement at low stimulation frequencies in model mice. At the end of the trial, the effect of pyridostigmine was only present at 20 Hz (*P =* 0.003) and absent at any of the other tested frequencies, suggesting a detrimental effect of pyridostigmine on neuromuscular transmission over time. At 12 weeks there was not a significant difference in decrement between combination therapy (pyridostigmine plus salbutamol) and pyridostigmine monotherapy at low or moderate stimulation frequencies. But, at high stimulation frequencies (70 and 100 Hz), decrement was significantly less in mice during combination therapy, compared to pyridostigmine monotherapy (*P =* 0.022 at 70 Hz and *P =* 0.015 at 100 Hz) (Fig. 3E and F). Salbutamol alone did not improve decrement.

### Salbutamol added to pyridostigmine ameliorates postsynaptic transmission in diaphragm preparations

**Figure 4:**
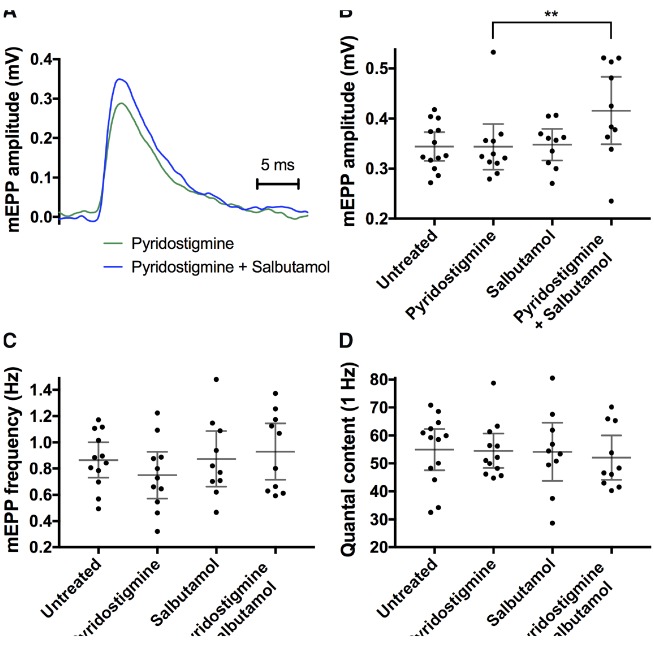
**MEPPs recorded from diaphragm preparations in model mice following the treatment protocol.** (**A**) Example trace of a mEPP of an animal treated with pyridostigmine monotherapy (green) and combination therapy of pyridostigmine and salbutamol (blue) (averaged from ∼30 captured events). (**B**) MEPP amplitudes; (**C**) mEPP frequency; (**D**) quantal content. Dot plots (**B**–**D**), with datapoints representing animal means, horizontal lines representing group means and error bars 95% CI (*n =* 10–13 mice per group; **P < 0.01 obtained from linear mixed model).

*In vivo* electromyography (decrement of CMAPs on repetitive nerve stimulation) suggests that neuromuscular transmission is enhanced by combination therapy. To obtain more detailed information of presynaptic and postsynaptic function of the neuromuscular junction, mEPPs and EPPs were recorded in phrenic nerve-diaphragm preparations, a standard muscle-nerve preparation for end-plate recording. Miniature end-plate potentials are attributed to the spontaneous release of one vesicle containing acetylcholine from the nerve terminal and binding to local postsynaptic AChRs, giving rise to a small depolarization. Example traces of mEPPs and EPPs in wild-type and model mice are displayed in Supplementary Fig. 2. Mean mEPP amplitude and frequency (0.34 mV and 0.87/s, respectively; Fig. 4) were lower in model mice than in wild-type mice (1.21 ± 0.23 mV and 1.94 ± 0.46/s, respectively, mean ± 95% CI, *n =* 6), reflecting lower AChR density at the postsynaptic membrane. End-plate recording in model mice following treatment is summarized in Fig 4. Representative mEPP traces in model mice following pyridostigmine monotherapy or combination therapy (pyridostigmine plus salbutamol) are shown in Fig. 4A. Significantly higher mEPP amplitudes were recorded following combination therapy in comparison to pyridostigmine monotherapy (*P =* 0.006), which might suggest a higher AChR density at the postsynaptic membrane (Fig. 4B). Treatment with pyridostigmine or salbutamol alone did not show significant changes to mEPP amplitudes. Frequency of mEPPs appeared lower in mice treated with pyridostigmine alone, but this reduction was not significant (Fig. 4C). Rise and decay times of mEPPs are illustrated in Supplementary Table 2. There was no significant difference in resting membrane potential that could have affected mEPP (or EPP) amplitudes (Supplementary Table 2). We did not measure muscle fibre size during diaphragm recordings (as it was not technically possible with the available equipment), but an increase in muscle fibre size *per se* would be unlikely to explain an increase in mEPP amplitude, as mEPP amplitude and muscle fibre size values are inversely related (Katz and Thesleff, 1957).

End-plate potentials were evoked by applying brief depolarizing pulses to the phrenic nerve, resulting in release of multiple neurotransmitter vesicles from the nerve terminal, and thus causing a large depolarization of the motor end-plate. The number of vesicles released during nerve stimulation, quantal content, was estimated from the division of EPP amplitudes (recorded at 1 Hz) by mEPP amplitudes. No differences in quantal content were present between treatment groups (Fig. 4D). In addition, no differences in EPP rundown at stimulation frequencies between 0.5 and 100 Hz were detected between treatment groups (data not shown). In conclusion, EPP recordings in phrenic nerve-diaphragm preparations did not indicate differences in stimulated synaptic vesicle release, at low or high frequencies.

### Salbutamol added to pyridostigmine increases postsynaptic area in fast-twitch muscles

**Figure 5:**
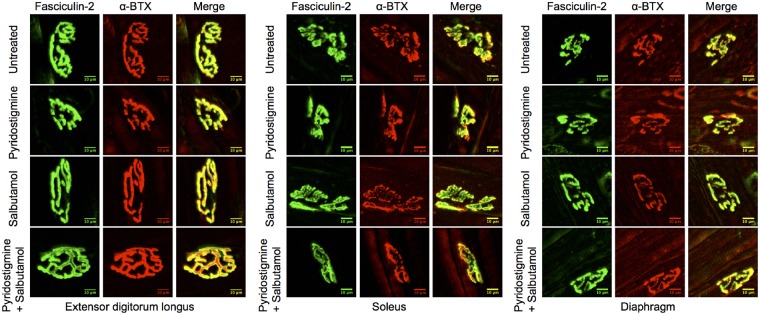
**Fluorescent staining of neuromuscular junctions in extensor digitorum longus, soleus and diaphragm muscles of model mice following treatment.** Representative images of neuromuscular junctions, stained with fasciculin-2 (green) and α-bungarotoxin (α-BTX, red, images were contrast enhanced for better visualization because of low expression level of AChR in model mice). Fasciculin-2 labels acetylcholinesterase (AChE), and α-bungarotoxin labels AChR. The yellow colour of the merged images represents co-localization of acetylcholinesterase and AChR. Scale bar = 10 μm.

Altered electrophysiological properties could arise from changes in postsynaptic structure. Changes at the neuromuscular junction structure were first assessed by staining with fluorescent markers. To be able to evaluate distinctive effects of drugs on different muscle types, neuromuscular junctions were stained in diaphragm muscles, extensor digitorum longus (as a prototype of fast-twitch muscle) and soleus muscles (as a prototype of slow-twitch muscle). The postsynaptic AChRs were visualized by fluorescent staining, using α-bungarotoxin (Alexa Fluor® 594). As AChR staining intensity was very weak in model mice, fasciculin-2 (Alexa Fluor® 488) was used as a second measure of neuromuscular junction location/size. Fasciculin-2 strongly stains acetylcholinesterase complexes (which co-localize with AChR staining; Peng *et al.*, 1999) in model mice. Example images of fluorescent staining of neuromuscular junctions in model mice are shown in Fig. 5. Neuromuscular junction areas in model mice were smaller than in wild-type mice, with results for wild-type mice summarized in Supplementary Fig. 3.

**Figure 6:**
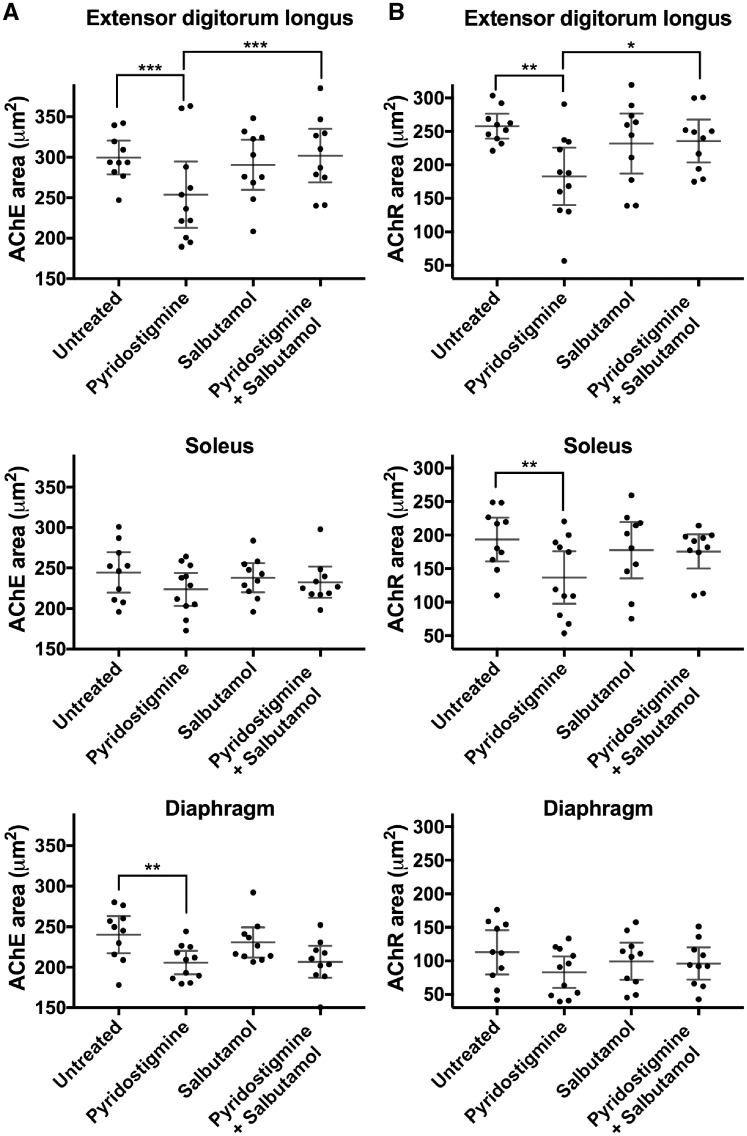
**Neuromuscular junction areas following fluorescent staining of extensor digitorum longus, soleus and diaphragm muscles in model mice following treatment.** (**A**) Neuromuscular junctions stained for acetylcholinesterase (AChE) (*left column*). (**B**) Neuromuscular junctions stained for AChR (*right column*). Dot plots with datapoints representing animal means, horizontal lines representing group means and error bars 95% CI (*n =* 10–11 mice per group; *P < 0.05, **P < 0.01, ***P < 0.001 obtained from linear mixed model). The smaller area for α-bungarotoxin staining reflects the loss of fluorescence intensity within the pretzel region due to the end-plate AChR deficiency.

Because of low AChR expression and thus low fluorescent intensity of α-bungarotoxin staining in model mice, measured neuromuscular junction areas were generally smaller for AChR than for acetylcholinesterase staining (Fig. 6A and B). Long-term treatment with pyridostigmine alone reduced neuromuscular junction areas stained for AChR and/or acetylcholinesterase in extensor digitorum longus, soleus and/or diaphragm muscles (*P <* 0.01). By contrast, combination therapy (pyridostigmine plus salbutamol) ameliorated this reduction in extensor digitorum longus muscles (*P <* 0.05). Salbutamol alone did not influence postsynaptic area. No difference in AChR pixel intensity or fragmentation between groups was found in any of the muscles (data not shown).

Potentially, changes to muscle fibre size or fibre type distribution might influence indirectly neuromuscular junction size. We therefore studied extensor digitorum longus and soleus muscles, as prototypes of fast- and slow-twitch muscles, respectively. No statistical differences in fibre type size or proportions were detected between groups, in any of the muscles examined (Supplementary Fig. 4). No positive correlation was found between muscle fibre size and neuromuscular junction size in model mice (Spearman correlation r = −0.250 and r = −0.295 for AChR and acetylcholinesterase staining in extensor digitorum longus muscles, respectively) (Supplementary Fig. 4C).

### Prolonged treatment with pyridostigmine reduces postsynaptic folding but is ameliorated by the addition of salbutamol

**Figure 7:**
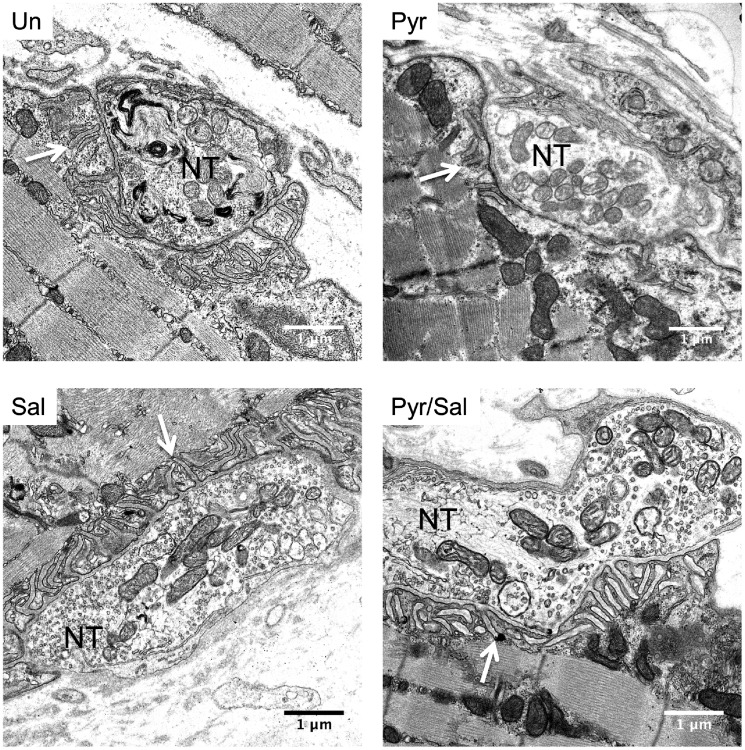
**Electron microscopic images of end-plate regions of extensor digitorum longus muscles of model mice at the end of the treatment protocol.** Representative ultrastructural images of end-plates. Arrows indicate postsynaptic folds. Scale = 1 μm. NT = nerve terminal; Pyr = pyridostigmine; Pyr/Sal = pyridostigmine + salbutamol; Sal = salbutamol; Un = untreated.

**Figure 8:**
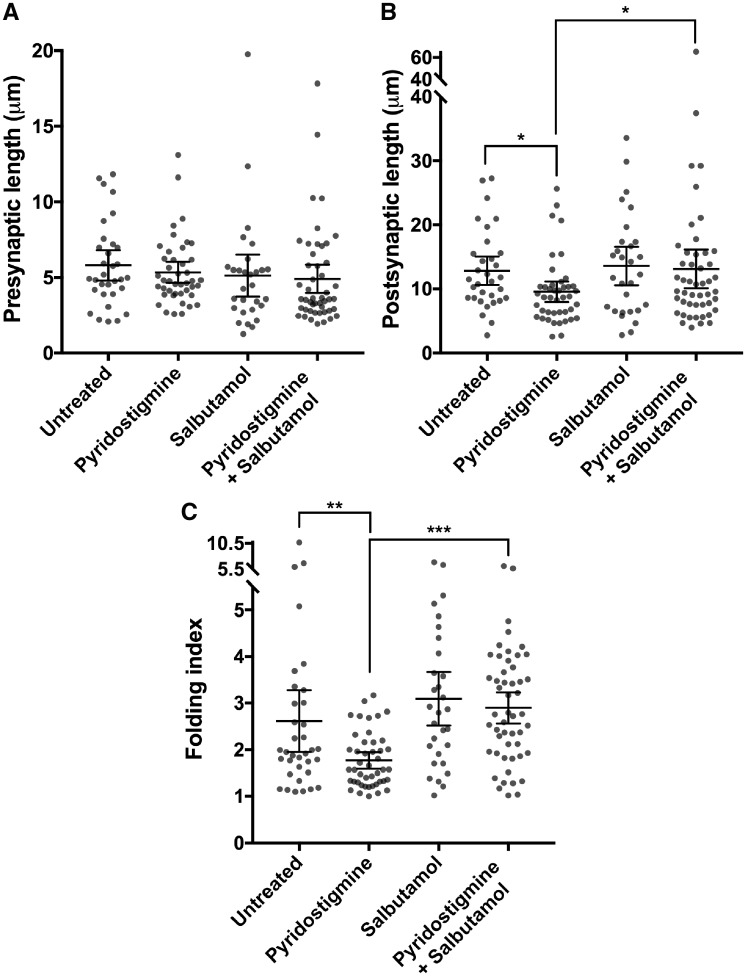
**Morphometric analysis of end-plate regions of extensor digitorum longus muscles of model mice at the end of the treatment protocol, studied by electron microscopy.** (**A**) Presynaptic length. (**B**) Postsynaptic length. (**C**) Folding index. Dot plots, with datapoints representing measurements, horizontal lines representing group means and error bars 95% CI (*n =* 32–53 end-plate regions of one to two mice per group; *P < 0.05, **P < 0.01, ***P < 0.001 obtained from linear mixed model).

Fluorescent staining under the light or confocal microscope may underestimate major changes to end-plate morphology through lack of resolution. We observed the greatest effect of drug treatment on postsynaptic area in the fast-twitch extensor digitorum longus muscles, and therefore chose these muscles for further investigation of postsynaptic parameters of end-plate regions by electron microscopy. Example ultrastructural images of end-plate regions of wild-type and AChR deficiency model mice are shown in Supplementary Fig. 5. As occurs in AChR deficiency syndrome in humans (Sieb *et al.*, 2000), the folding index of the postsynaptic folds was markedly reduced in model mice (mean 2.62; Fig. 8) compared to wild-type mice (6.12 ± 2.38, mean ± 95% CI). Typical ultrastructural images of end-plate regions of model mice following treatment are shown in Fig. 7. Measurements of ultrastructural pre- and postsynaptic features in model mice following treatment are displayed in Fig. 8. Presynaptic length was not significantly different across groups. However, treatment with pyridostigmine alone significantly reduced both postsynaptic length (*P =* 0.02) and folding index (*P =* 0.009). This reduction in postsynaptic length and folding index caused by long-term pyridostigmine was rescued (*P =* 0.035 and *P <* 0.001, respectively) by combining salbutamol with pyridostigmine. Salbutamol alone did not statistically significantly enhance the folding index, when compared to untreated mice.

## Discussion

In this study we show that the beneficial response to β2-adrenergic agonists of patients with AChR deficiency due to ε-subunit mutations on long-term anticholinesterase medication is sustained over time. In a disease model, we find that salbutamol ameliorates the detrimental effects on postsynaptic structure and thus on function that occur as a result of prolonged therapy with pyridostigmine. The data are supported by congruent results of motor behavioural tests, as well as detailed functional and morphological tests of the neuromuscular junction.

Onset of symptoms for AChR deficiency due to mutations in the ε-subunit is usually in the neonatal period or in early childhood. The disease course is usually stable throughout life, as observed in our mouse model. Distribution of muscle weakness in AChR deficiency is diffuse, and includes limb and respiratory muscles. In our previous report (Rodriguez Cruz *et al.*, 2015) we restricted our study cohort to more severely affected cases (QMG score ≥15), whereas here we included three less severely affected individuals (QMG scores 11, 9, 9), and in these cases also found improved QMG severity scores after adding salbutamol to their medication. It was frequently a deterioration in strength, in keeping with potentially harmful long-term effects of acetylcholinesterase inhibitors on neuromuscular junction structure and function (Chang *et al.*, 1973; Engel *et al.*, 1973; Gillies and Allen, 1977) and our own observations that led us to initiate β2-adrenergic agonist treatment. Our disease model demonstrates that salbutamol can ameliorate these adverse effects on neuromuscular junction structure, and the response to respective treatments seen in this model reflects closely the response to treatment experienced in our patient cohort. We have noted that the beneficial response to the addition of salbutamol tends to be faster in congenital myasthenic patients with AChR deficiency than is seen for congenital myasthenic patients with DOK7 mutation (Lashley *et al.*, 2010), generally over a period of weeks rather than months, and this was also reflected in the relatively quick response to the addition of salbutamol seen in the mouse model.

In the AChR deficiency mouse model prolonged pyridostigmine treatment reduced postsynaptic areas, as examined by fluorescent staining. Adding salbutamol medication to the pyridostigmine-treated animals increased postsynaptic areas in extensor digitorum longus muscles (Fig. 6). The absence of a clear response to combination therapy in the other muscles is thought to be related to the less apparent detrimental effect of pyridostigmine in soleus and diaphragm muscles in our study. Fluorescent staining of the postsynaptic neuromuscular junction is likely to underestimate the true extent of postsynaptic surface changes, and so we investigated the end-plates by electron microscopy in extensor digitorum longus muscles. Analysis of electron microscopic end-plate images from extensor digitorum longus muscles showed profoundly reduced postsynaptic length and folding in pyridostigmine treated mice, compared to untreated mice. The rescue of this loss of postsynaptic folds by adding salbutamol was the most notable change we observed in neuromuscular junction structure. Possibly, the effect of β2-adrenergic agonists on postsynaptic folding might be even more pronounced in humans than rodents, as throughout evolution the extent of postsynaptic folding dramatically increased (Slater, 2017). It has been estimated that the effect of the postsynaptic folds and the positioning of the voltage-gated sodium channels within them is to double the safety factor for neuromuscular transmission (Wood and Slater, 1997) and thus the enhanced folding seen is in keeping with the therapeutic response. Our morphological findings are supported by a recent study in a transgenic mouse model of acetylcholinesterase deficiency (McMacken *et al.*, 2019).

The functional effect of combination therapy on neuromuscular transmission was explored in the AChR deficiency model. Adding salbutamol to pyridostigmine reduced decrement of CMAPs on electromyography in our study, compared to pyridostigmine alone, but at high nerve stimulation frequencies only. This may be due to acetylcholinesterase inhibitors being least effective at high nerve stimulation frequencies (Tiedt *et al.*, 1978; Chang *et al.*, 1986) and therefore the beneficial effects of combination therapy on impaired neuromuscular transmission being more easily detected. Improvement in decrement reflects an increase in safety factor of neurotransmission, which is crucial at high nerve stimulation frequencies, when acetylcholine release can be reduced (Wood and Slater, 1997, 2001). This observation might have clinical relevance for fast-twitch muscles, where nerve stimulation frequencies of up to 100 Hz can be reached, usually in short bursts of 5–10 stimuli (Hennig and Lomo, 1985). As high nerve stimulation frequencies are considered less reliable than low nerve stimulation frequencies, further studies will be required to ascertain this observation.

In contrast to our study, previous similar studies of long-term anticholinesterase medication have managed to detect a reduction in mEPP amplitudes, but these were generally performed in healthy rodents or using higher levels of cholinesterase inhibitors (Engel *et al.*, 1973; Ward *et al.*, 1975; Gillies and Allen, 1977; Morsch *et al.*, 2013). It is thought that this is caused by a reduction of AChR sites as a result of diminished folding of the postsynaptic membrane that occurs with long-term use of acetylcholinesterase inhibitors (Chang *et al.*, 1973; Engel *et al.*, 1973; Ward *et al.*, 1975). Since amplitudes of mEPPs were low in AChR-deficient mice and close to the limits of detection, reflecting the low levels of AChR in the postsynaptic membrane, we assume that a proportion of the smallest mEPPs was undetectable within recording noise (Plomp *et al.*, 1992; Cossins *et al.*, 2004). This is in accord with the observation of lower mEPP frequency in pyridostigmine-treated mice. However, we were able to detect a significant increase in mEPP amplitudes following combination therapy, compared to pyridostigmine monotherapy. An increase in mEPP amplitude is thought most likely to correspond to an increase in density of postsynaptic AChR channels (Plomp *et al.*, 2015; Slater, 2017). This likely increase in AChR density might have been too subtle to detect by fluorescent staining.

The molecular mechanism through which β2-adrenergic agonists work at the neuromuscular junction structure is still not clear. However, our study and studies by others (Webster *et al.*, 2013; McMacken *et al.*, 2019) show that the effect is unlikely to be related to muscle hypertrophy or muscle fibre transition in neuromuscular junction disorders (Supplementary Fig. 4), but is related (at least in part) to a direct effect at the neuromuscular junction. Myotube cultures harbouring DOK7 mutations demonstrate that β2-adrenergic agonists increased AChR clustering and slowed AChR cluster dispersal (Clausen *et al.*, 2018), suggesting an effect on postsynaptic muscle β2-adrenoreceptors. Zebrafish with impaired MuSK function show that β2-adrenergic agonists improved neuromuscular junction morphology (McMacken *et al.*, 2018). Chemical sympathectomy results in disruption of pre- and postsynaptic structures, and disruption is rescued by β2-adrenergic agonists (Khan *et al.*, 2016; Rodrigues *et al.*, 2015). As salbutamol alone did not result in significant changes in electrophysiological or morphological parameters of neuromuscular junctions in this study, disturbance of the balance between the agrin-induced AChR clustering pathway and synaptic disruption due to prolonged presence of acetylcholine during neuromuscular transmission might be a prerequisite for salbutamol to have an overt beneficial effect on the neuromuscular junction structure. Mice treated with salbutamol alone, however, did show a modest improvement on the hang test, which might have been related to improved muscle contraction.

The experiments presented here model treatment for AChR deficiency due to *CHRNE* mutations. They show the marked benefit of anticholinesterase medication, but also, in keeping with a series of studies from the 1970s (Engel *et al.*, 1973; Gillies and Allen, 1977) demonstrate that there can be longer term adverse effects on neuromuscular junction structure. Adding β2-adrenergic agonists in the treatment of this disorder can counter the adverse effects on synaptic structure and signal transmission. Gauging the balance between (i) the AChR clustering pathway; and (ii) synaptic disruption induced by prolonged presence of acetylcholine during neurotransmission has already formed an empiric basis for determining treatment strategy for many other forms of congenital myasthenic syndromes (Beeson, 2016). Our study gives further scientific support for the strategic use of the two main drugs in congenital myasthenia. The results of this study have practical implication for the treatment of patients with autoimmune myasthenia gravis on long-term anticholinesterase medication (Soliven *et al.*, 2009; Lipka *et al.*, 2017) and also for other neurological conditions (Gonzalez-Freire *et al.*, 2014; Bartus *et al.*, 2016; Pera *et al.*, 2018) in which a role for the neuromuscular junction is implicated.

## Supplementary Material

awz322_Supplementary_Material

## Abbreviations

AChR =: acetylcholine receptor
CMAP =: compound muscle action potential
(m)EPP =: (miniature) end-plate potential
QMG score =: Quantitative Myasthenia Gravis score
