# Supplementary material for: β2-Adrenergic receptor agonists ameliorate the adverse effect of long-term pyridostigmine on neuromuscular junction structure
Source: Brain. 2019 Oct 21;142(12):3713–27. doi: 10.1093/brain/awz322 (PMC6892641; doi:10.1093/brain/awz322)
Supplement: awz322_Supplementary_Material [file awz322_supplementary_material.pdf]

Supplementary material

Supplementary Figure 1. Assessment of body weight during treatment protocol. Arrows indicate drops in body weight, which occurred after anaesthetic events. Data points and error bars represent group means ± 95% C.I. (n=10-13 mice per group; ns, not significant based on Linear mixed model).

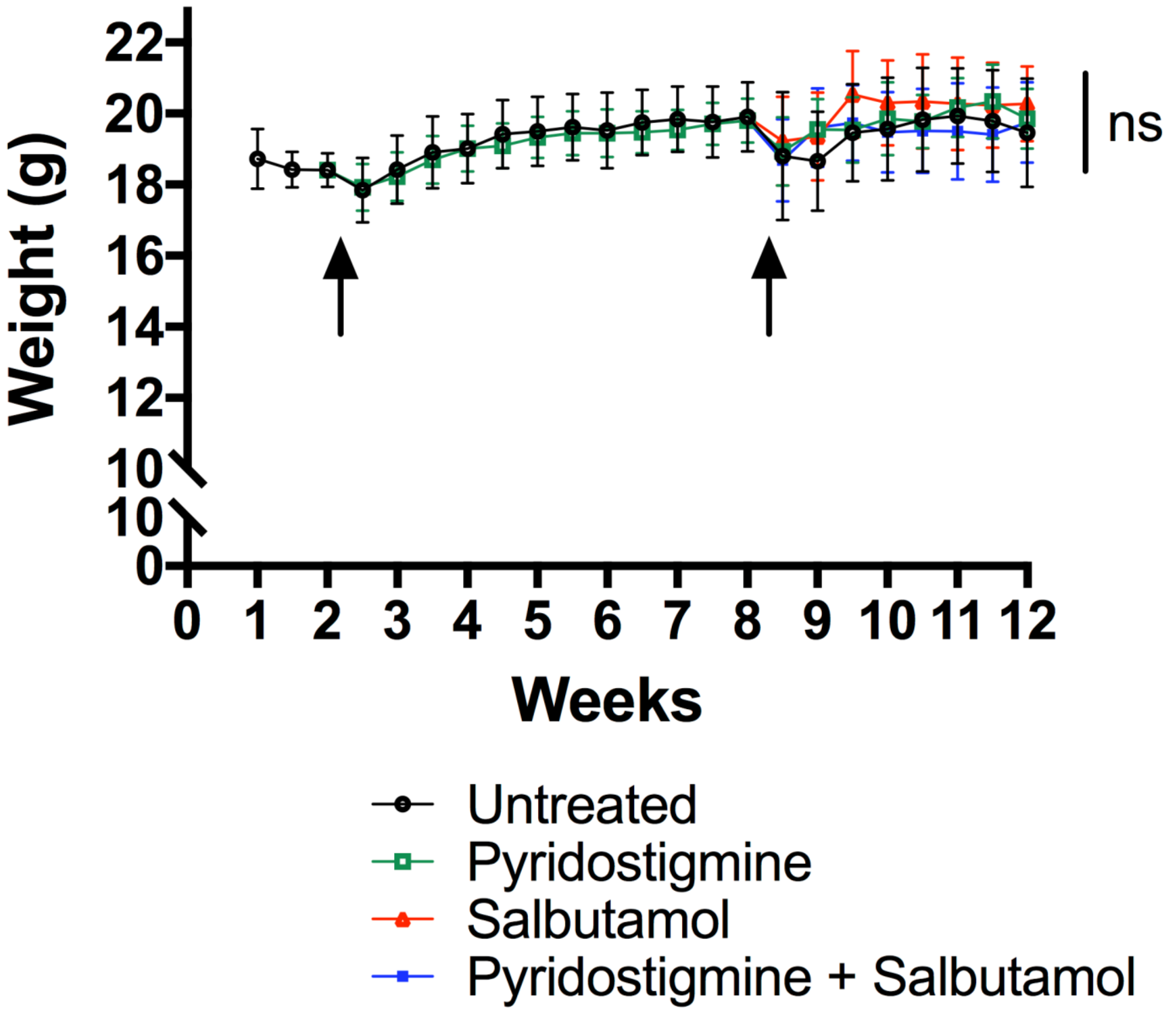

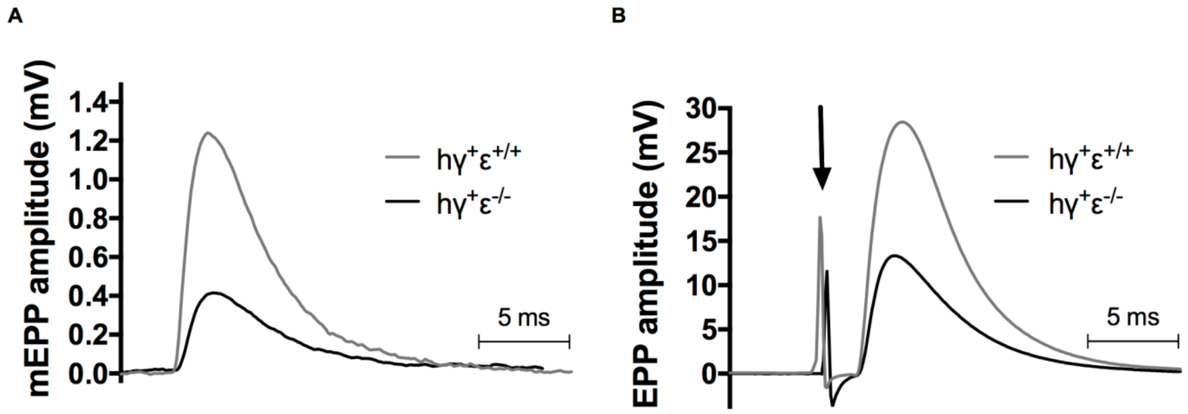

Supplementary Figure 2. Recording traces of miniature endplate potentials (mEPPs) (A) and endplate potentials (EPPs) (B) from phrenic nerve-hemidiaphragm preparations of wildtype (h$\gamma^{+}\varepsilon^{+/+}$) and model (h$\gamma^{+}\varepsilon^{-/-}$) mice. The arrow indicates the stimulation artefact. Amplitudes are lower and decay times longer for mEPPs and EPPs in model mice. The longer decay time is due to the longer open times of the $\alpha_2\beta\gamma\delta$ AChR channel, in comparison to $\alpha_2\beta\varepsilon\delta$ channel (Gu *et al.*, 1990).

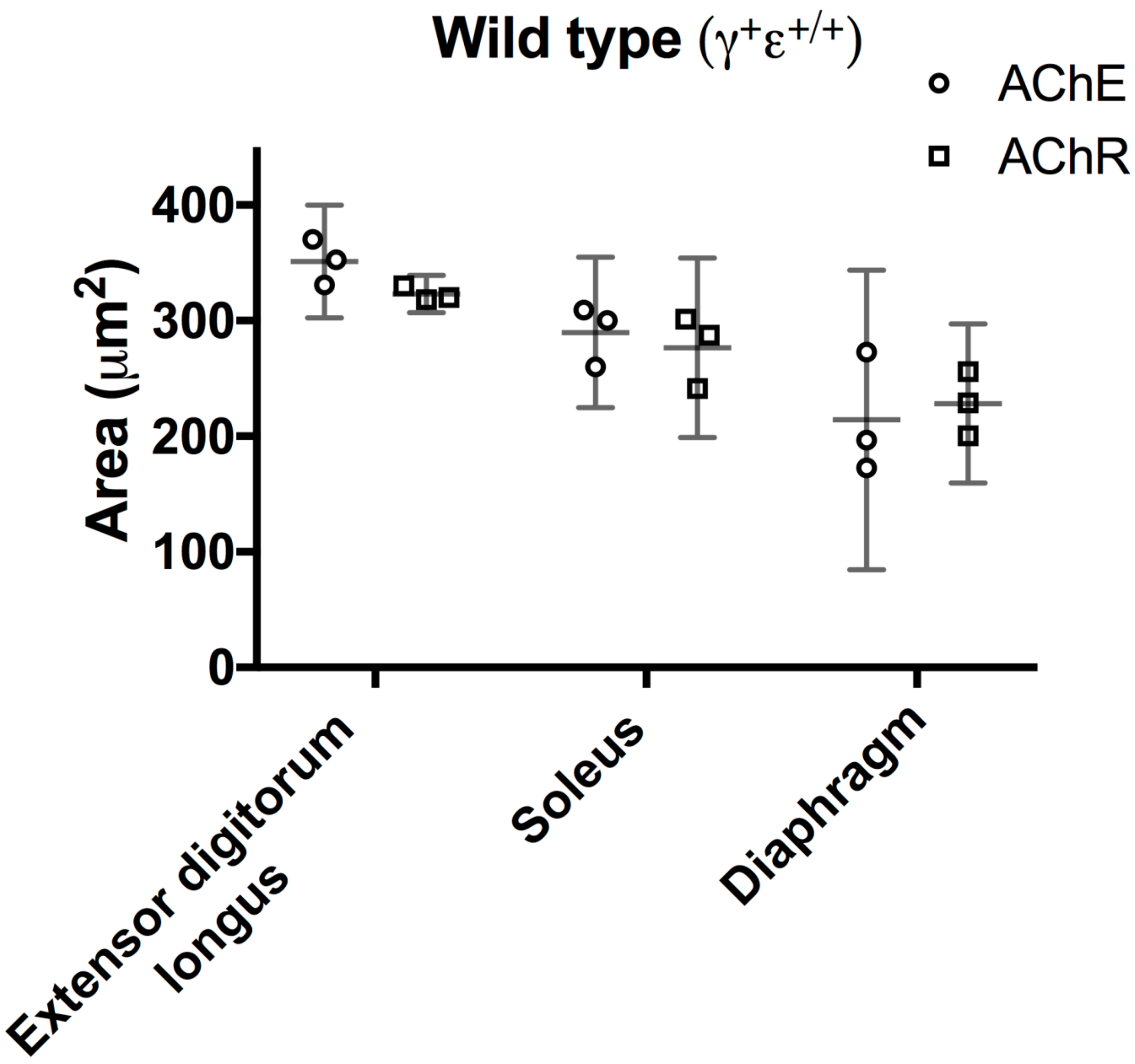

Supplementary Figure 3. Wildtype (h$\gamma^{+}\varepsilon^{+/+}$) data for fluorescent staining of neuromuscular junctions in extensor digitorum longus, soleus and diaphragm muscles. Areas of neuromuscular junctions stained for acetylcholinesterase (AChE) and AChR. Dot plot, with data points representing animal means, horizontal lines representing group means and error bars 95% C.I.

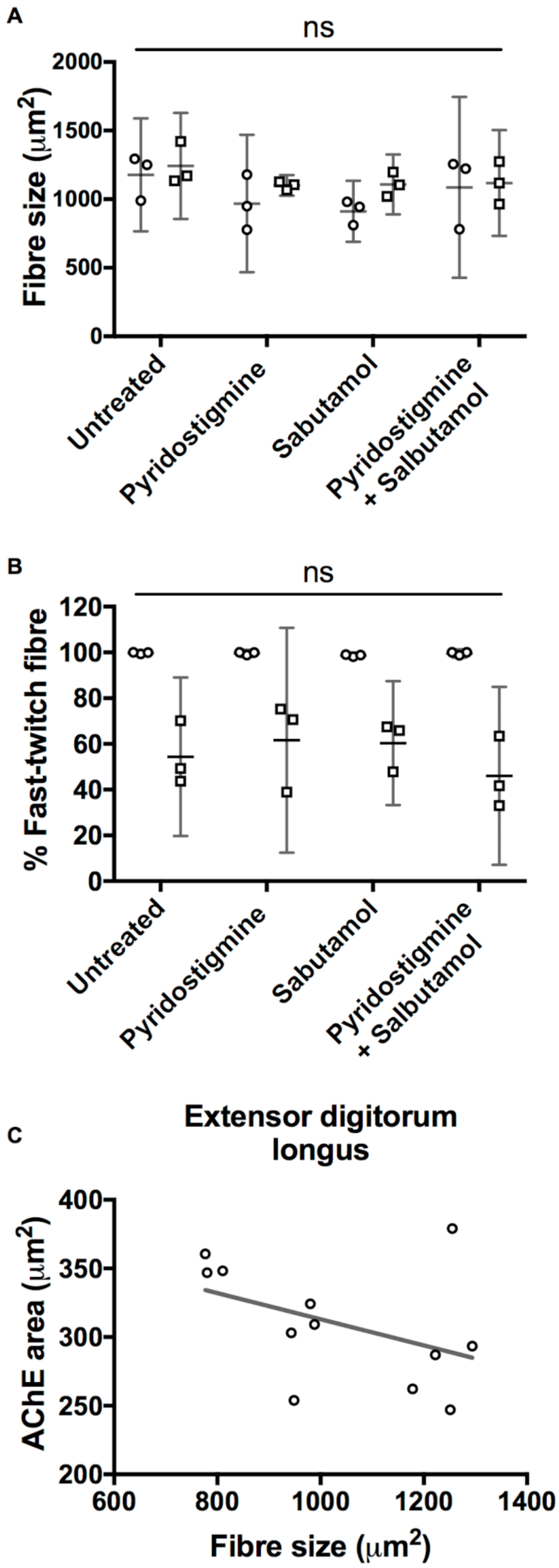

Supplementary Figure 4. Muscle fiber size and fiber type distribution in extensor digitorum longus and soleus muscles in model mice following treatment. A. Muscle fiber size **B.** Fiber type distribution, presented as % fast-twitch type. Dot plots (*A*-*B*), with data points representing animal means, horizontal

lines representing group means and error bars 95% C.I. No significant differences in fibre type size or distribution were detected between treatment groups. (n=3 mice per group; ns, not significant based on Binomial Mixed Model). **C.** Scatter plot, describing relationship between muscle fibre size and acetylcholinesterase (AChE) area in extensor digitorum longus muscles, with data points representing animal means. There was no positive correlation between muscle fibre size and acetylcholinesterase area. (n=12 mice; ns, not significant based on Spearman correlation).

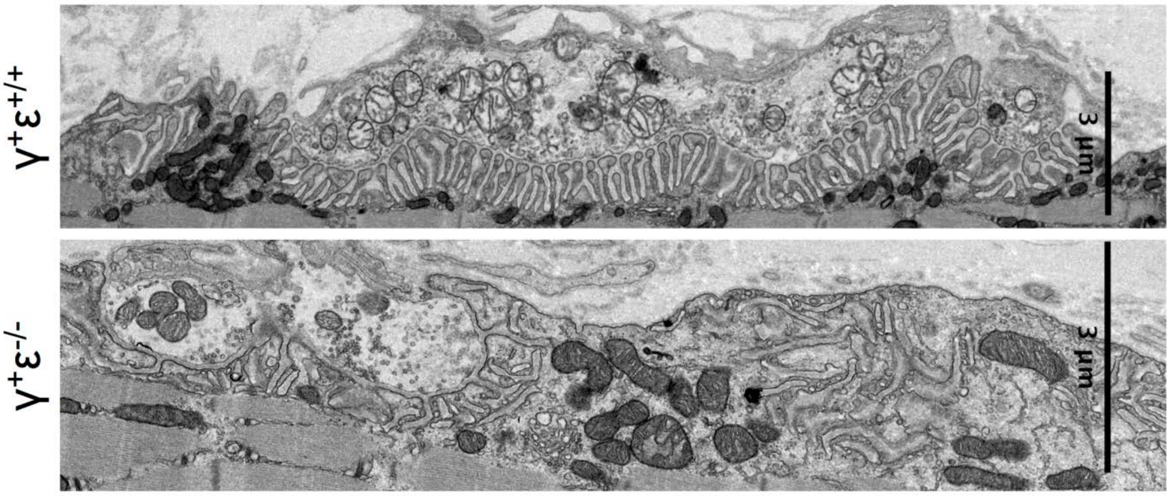

Supplementary Figure 5. Representative electron microscopic images of endplate regions of extensor digitorum longus muscles. A. From a wild type (h$\gamma^{+}\varepsilon^{+/+}$) mice. **B.** From an AChR deficiency model (h$\gamma^{+}\varepsilon^{-/-}$) mouse**.** Scale = 3 µm. Note, mitochondria appear swollen in some ultrastructural images (here in top panel), this is most likely due to slight delay in fixation.

Patient	Kinship	Mutation	Acetylcholinesterase inhibitor			β2-adrenergic agonist	
			Pyridostigmine (mg/day)	Neostigmine (mg/day)	3,4-DAP (mg/day)	Salbutamol (mg/day)	Ephedrine (mg/day)
1	1	c.614_620del7 (p.Trp205fs)<br>c.918-1G>A	225	-	20	8	-
2	1	c.614_620del7 (p.Trp205fs)<br>c.918-1G>A	300	-	50	12	
3	2	c.614_620del7 (p.Trp205fs)<br>c.614_620del7 (p.Trp205fs)	120-180	-	40-60	12	-
4	2	c.614_620del7 (p.Trp205fs)<br>c.614_620del7 (p.Trp205fs)	300	-	50	12	-
5	3	c.972+1G>T *<br>c.972+1G>T	360	-	80	8	-
6	4	c.1267-32_1319del<br>c.1267-32_1319del	120-180	-	-	-	30
7	5	c.1033-1G>C<br>c.1353dupG (p.Asn452fs)	300	-	-	12	-
8	6	c.1267delG (p.Glu423fs)<br>c.1267delG (p.Glu423fs)	120-180	-	30	6	-
9	7	c.553del7<br>c.911delT	300	-	40	6	-
10	1	c.614_620del7 (p.Trp205fs)<br>c.614_620del7 (p.Trp205fs)	-	60	-	8	
11	8	c.650G>T (p.R217L)<br>c.833_835delCTC (p.S278del)	120	-	-	8	-

*****This variant would be called c.1032+1G>T using HGVS nomenclature

Supplementary Table 2. Miniature endplate potential (mEPP) rise / decay time and resting membrane potential recorded from phrenic nerve-hemidiaphragm preparations in model mice.

**Treatment group**	**mEPP rise time (ms)**	**mEPP decay time (ms)**	**Resting membrane potential (mV)**
Untreated	1.77 (1.70-1.83)	9.98 (9.63-10.33) ¶	-67.59 (-68.22, -66.96)
Pyridostigmine	1.98 (1.92-2.05)	10.17 (9.76-10.58)	-67.97 (-68.63, -67.32)
Salbutamol	1.85 (1.76-1.94)	9.23 (7.00-11.46) ¶	-67.94 (-68.61, -67.28)
Pyridostigmine + Salbutamol	1.89 (1.82-1.96)	9.52 (9.15-9.89)	-69.57 (-70.23, -68.90)

Data is expressed as means ± 95% C.I. (n=10-13 mice per group; ¶$P<0.01$ obtained from Linear mixed model).
